# Effect of Drynaria rhizome in-situ gel in periodontitis management

**DOI:** 10.6026/973206300221910

**Published:** 2026-03-31

**Authors:** Kaushal Pati Tripathi, Neelam Mittal, Anju Gautam, Aishwarya Pandey, Sakshi Gupta, Rashika M

**Affiliations:** 1 Department of Periodontology, Faculty of Dental Sciences, Institute of Medical Sciences, Banaras Hindu University, Varanasi, India; 2 Department of Conservative Dentistry and Endodontics, Faculty of Dental Sciences, Institute of Medical Sciences, Banaras Hindu University, Varanasi, India

**Keywords:** Periodontitis, local drug delivery system (LDDS), Dryanria, herbal gel

## Abstract

The effect of herbal in-situ gel in managing chronic periodontitis is of interest. Therefore, one such herb, Drynaria quercifolia Rhizome in-situ gel was compared with chlorhexidine gel for the treatment of chronic periodontitis. Hence, a total of eighty four sites with probing pockets ≥5 mm were randomly assigned to either gel following scaling or root planing. Clinical parameters-Plaque Index (PI), Gingival Index (GI), Probing Pocket Depth (PPD) and Clinical Attachment Level (CAL) were recorded at baseline, 1 month and 2 months. Both groups showed significant improvements, with the Drynaria gel demonstrating greater PPD reduction at 2 months (p < 0.001) despite slightly higher PI and GI scores. No adverse effects were noted, suggesting Drynaria gel is a safe and effective natural adjunct, warranting confirmation in larger, long-term studies.

## Background:

Periodontal diseases encompass a range of inflammatory conditions affecting the supporting structures of the teeth, including the gingiva, periodontal ligament, cementum and alveolar bone [1]. They pose a significant global health burden, with severe forms leading to tooth loss and systemic complications [2]. Periodontitis initiates as gingivitis and, if untreated, advances to involve deeper structures, leading to pocket formation and growth of anaerobic microorganisms such as Porphyromonas gingivalis, Prevotella intermedia, Aggregatibacter actinomycetemcomitans and Fusobacterium nucleatum [3]. Conventional treatment includes mechanical debridement like scaling, root planing and flap surgery [4]. However, these cannot eliminate all microorganisms due to their deep penetration in tissues [5]. Thus, adjunctive antimicrobial agents are essential an approach termed Perioceutics [6]. Systemic antibiotics face limitations due to side effects and microbial resistance [7]. Local Drug Delivery Systems (LDDS) offer targeted drug application in pockets, enhancing efficacy and minimizing systemic exposure [8]. The placement of local drug delivery systems (LDDS) is in fact facilitated by the presence of periodontal pockets, which act as a natural reservoir [9]. The gingival crevicular fluid (GCF) within the pockets provides a humid environment that enhances the dispersion of the drug throughout the pocket over a sustained period of time fostering its efficacy [10]. In the pursuit of novel and natural treatment options, the exploration of indigenous Ayurvedic medicines has become increasingly relevant [11]. Among natural alternatives, Drynaria quercifolia (Oak-Leaf Fern) has shown antimicrobial, anti-inflammatory and bone-regenerative potential due to flavonoids, phenolic acids and triterpenoids [12, 13]. Rhizome extracts of this plant have shown to improve bone cell viability and enzymatic markers and the leaves possess anti-inflammatory and analgesic properties and are used in the form of poultices to relieve pain and swelling [14]. Therefore, it is of interest to evaluate and compare the efficacy of a Drynaria in-situ gel with chlorhexidine in-situ gel as a local drug delivery agent in the treatment of periodontal diseases, contributing to the advancement of natural and sustainable therapeutic approaches for thisprevalent oral health condition.

## Materials and Methods:

This randomized clinical trial was approved by the Institutional Ethics Committee (ECR/526/Inst/UP/2014/RR-20), registered with the Clinical Trials Registry- India (CTRI/2024/05/066873) and conducted in accordance with the Declaration of Helsinki. Written informed consent was obtained from all participants after a detailed explanation of the study procedures, risks and benefits. The inclusion criteria included systemically healthy patients diagnosed as having chronic periodontitis with periodontal pockets of ≥5 mm, no periodontal treatment received in the past 6 months or radiotherapy, no use of antimicrobial medications in the past 3 months. Patients with unacceptable oral hygiene during the study period, smokers, tobacco chewers, pregnant women and lactating mothers were excluded. The primary outcome measures of the study were probing pocket depth and clinical attachment level. Secondary outcome measures included plaque index and gingival index. The sample size was calculated based on the study by, Vijay *et al.* 2022 keeping the power 95% alpha 0.05% the effect size of 0.79, the sample size came out to be 42 per group, (n = 84) sample size was calculated using GPS software (3.1.9.7) [15].

Thus the study participants were divided into two groups with forty two participants in each,

[1] Group A: Dry aria in-situ gel (Test)

[2] Group B: Chlorhexidine in-situ gel (Control)

A comprehensive assessment of each patient's oral hygiene and periodontal health was done prior to commencement of therapy. Afterward, scaling and root planning procedures were done in each recruited patient. In addition, patients received personalized oral hygiene instructions to help improve and maintain their oral health. Following scaling and root planing; the site was irrigated with normal saline. In patients of Group A, Dry aria in-situ gel was carefully placed into the gingival sulcus, filling it up to the level of the gingival margin with an applicator tip. After this, Coe-pack periodontal dressing was applied to ensure protection of the treated site shielding the area from external irritants for 1 week. Group B patients received chlorhexidine in-situ gel in the same manner. Meanwhile, all the subjects were instructed to avoid brushing their teeth till the removal of the Coe-pack dressing. Patients were recalled on the 7th day for removing Coe-pack. Also, oral hygiene instructions were given to the patients wherever required. All the clinical parameters were re-evaluated at one month and two months. The data of all the parameters included in the present study were assembled in Microsoft Excel 2021 and put through statistical analysis using IBM SPSS Statistics version 25. The Mann- Whitney U test and Independent t-test were employed for comparisons between groups. Friedman test for Intra-group changes. Post-hoc analysis was done using the Pairwise Friedman Two-way ANOVA test. In the above-mentioned tests, p ≤ 0.05 was considered statistically significant.

## Results:

A total of 84 patients with chronic periodontitis were included and evenly distributed into two treatment groups. Group A received scaling and root planing (SRP) followed by the application of dry aria in-situ gel, while Group B received SRP followed by Chlorhexidine (CHX) in-situ gel. Throughout the trial, there were no patient discomfort or complaints were obtained regarding the application of the in-situ gel. Clinical parameters including Gingival Index (GI), Plaque Index (PI), Probing Pocket Depth (PPD) and Clinical Attachment Level (CAL) were recorded at baseline, 1 month and 2 months post-treatment. Both groups demonstrated a significant improvement in gingival health, as evidenced by a reduction in GI scores from baseline to 1 month (p < 0.001). At the 2-month follow-up, the GI in the CHX group continued to decline (0.98 ± 0.13), whereas the Drynaria group showed a slight increase (1.13 ± 0.14), leading to a statistically significant intergroup difference in favor of CHX (p = 0.00) (Table 1 - see PDF). Similarly, the plaque accumulation as measured by the PI reduced significantly in both groups at 1 month (p < 0.001). Although a slight increase in PI was noted in both groups at 2 months, CHX-treated sites maintained slightly better plaque control (1.08 ± 0.14) than the Drynaria group (1.13 ± 0.12). However, this intergroup difference was not statistically significant (p = 0.05) (Table 2 - see PDF). Probing Pocket Depth (PPD) also showed a statistically significant reduction within both groups over the study period (p < 0.001). In the Drynaria group, PPD decreased from 5.52 ± 0.91 mm at baseline to 3.26 ± 0.82 mm at 2 months, whereas the CHX group showed a reduction from 5.38 ± 0.53 mm to 3.81 ± 0.59 mm. The intergroup comparison at 2 months indicated a statistically significant greater reduction in PPD for the Drynaria group (p < 0.001) (Table 3 - see PDF). Regarding Clinical Attachment Level (CAL), both treatment modalities resulted in substantial gains. The Drynaria group exhibited a CAL gain from 5.40 ± 1.01 mm at baseline to 3.90 ± 1.05 mm at 2 months, while the CHX group showed improvement from 5.16 ± 0.62 mm to 3.83 ± 0.76 mm. However, the intergroup differences at all-time points remained statistically insignificant (p > 0.001), suggesting that both agents were similarly effective in promoting clinical attachment gain (Table 4 - see PDF). Analysis of intragroup changes from baseline to 1 and 2 months (Table 5 - see PDF) confirmed statistically significant improvements (p < 0.001) in all four clinical parameters from baseline to 2 months within both groups, supporting the effectiveness of both treatments over time.

## Discussion:

Since ancient times, Drynaria quercifolia (L.) J.Sm. has been known to possess anti-inflammatory and antipyretic properties, as well as analgesic properties [16]. Additionally, it prevents typhoid fever; skin issues, bacterial infections and helps in combating appetite loss. It also works well for rheumatic pain, headaches, body aches and worm infestations [17]. Furthermore, a study has also investigated the effectiveness of Drynaria quercifolia plant (rhizome) extract as a bone-forming substitute in treating periodontal intraosseous defects [18, 19]. The objective of the current study was to clinically evaluate the efficacy of Drynaria quercifolia in comparison with chlorhexidine, sothat it can potentially be used as a local drug delivery (LDD) agent in treating periodontal diseases, providing a natural and sustainable therapeutic alternative against periodontal illness. The mean pH of periodontal pockets is 7.09. The rhizome extract of Drynaria quercifolia has been shown to speed up the synthesis of proteoglycans and raise alkaline phosphatase (ALP) activity, with little fluctuation depending on the depth of the pocket [20]. The mean pH of the drug-containing gel in our study was 6.4; hence there would be no interference with drug release or local irritation [21]. Our study focused on four main clinical outcomes: Plaque Index, Gingival Index, Probing pocket depth and Clinical Attachment Level (CAL). In both groups, every periodontal parameter showed an overall significant improvement. Behal *et al.* evaluated the impact of a 2% whole turmeric gel-based local drug delivery system as an adjunct to scaling and root planing (SRP) [22]. Their findings demonstrated a statistically significant improvement in various periodontal measures, including the gingival index (GI), plaque index (PI), sulcus bleeding index (SBI) and probing pocket depth (PPD). The results observed in our study showed that plaque accumulation significantly decreased within both groups, confirming their efficacy in plaque control. In the Drynaria group, PI reduced from 1.25 ± 0.22 at baseline to 0.97 ± 0.17 at one month and 1.13 ± 0.12 at two months. There is an increase in PI from one to two months (p = 0.001). Similarly, the Chlorhexidine group also showed a reduction in the PI at 1 month and increased at 2 months with respect to 1 month. Notably, the PI differences between the groups were not statistically significant at any time point, suggesting comparable plaque control efficacy between the two gels. In 2021, Saini *et al.* performed a clinical study to assess the effectivenessof Neem and Turmeric chips in patients with chronic periodontitis [23]. Similar to our findings, their results showed that both herbal treatments effectively reduced clinical parameters, including plaque index (PI), gingival index (GI) and probing pocket depth (PPD). In the Drynaria group, GI reduced to baselines to one month and then increased. The increase in GI between one and two months suggests that there might be a requirement for periodic reapplication of Drynaria gel for prolonged efficacy. On the other hand, the Chlorhexidine group exhibited a continuous reduction in GI, from 0 to 1 month, with significant changes from baseline to 1 month and non-significant- 1 month to 2 months of intervals. Intergroup comparisons revealed no significant difference at baseline and one month (p = 0.38); however, the difference became significant at two months (p = 0.00), favoring Chlorhexidine gel. Our results align with a similar study by Warad *et al.* which used 2% lemongrass oil gel in periodontitis patients and found favorable results in periodontal indices [24]. Reduction in PPD is a key clinical parameter in evaluating periodontal therapy outcomes. Both Drynaria and Chlorhexidine in-situ gels showed significant reductions in PPD over the study period (p < 0.001). The continued improvement from one to two months (p = 0.00) suggests the sustained efficacy of Drynaria gel in promoting periodontal healing, over the CHX gel in which a non-significant reduction from 1 month to 2 months has been observed. Intergroup comparisons revealed an improvement in PPD at two months (p = 0.00), with Drynaria gel showing superior improvement, potentially due to its bioactive properties that support tissue healing. The findings also accord with the research conducted by Katariya and Rajasekar which demonstrated that the clinical attachment level and probing depth were improved when aloe vera gel and SRP were combined [25]. CAL gains are critical for evaluating periodontal regeneration. In the Drynaria group, CAL improved significantly. Notably, the improvement continued from one to two months (p = 0.023), reflecting sustained periodontal regeneration. Similarly, the Chlorhexidine group exhibited significant CAL gains. However, the improvement between one and two months was less pronounced (p = 0.042), suggesting a tapering effect. Intergroup analysis showed no significant differences in CAL at any time point (p > 0.05), indicating comparable outcomes in attachment level improvement. Raghava *et al.* assessed the efficacy of curcumin gel as a local drug delivery (LDD) system alongside scaling and root planing (SRP), observing comparable improvements in plaque index, gingival index, pocket depth and clinical attachment level [26]. Similarly, Singh *et al.* compared the effectiveness of curcumin and chlorhexidine chip LDD systems in periodontal pockets [27]. Their results showed significant improvements in all periodontal parameters in both groups, suggesting that the curcumin chip could be a viable alternative to the chlorhexidine chip LDD system. Chlorhexidine remains a widely studied compound in the realm of oral health. Multiple clinical trials, including those by Van Strydonck *et al.* and James *et al.* have confirmed chlorhexidine's efficacy in controlling plaque and gingival inflammation [28, 29]. In this study, chlorhexidine gel showed significant improvements in all clinical parameters, consistent with its established role in periodontal therapy. The results of our study are encouraging, as it indicates that Drynaria in-situ gel can be a plant-based, biocompatible alternative to chlorhexidine.

## Conclusion:

We show the therapeutic potential of Drynaria rhizome as an innovative, economical with potential against periodontitis. However, while the results are encouraging, further research is warranted. Future studies with larger populations, diverse clinical scenarios and extended follow-up periods are necessary to comprehensively evaluate the long-term effectiveness, safety and cost-effectiveness of Drynaria gel. If validated, this novel formulation could mark a significant advancement in periodontal therapy and patient care.

## References

[1] Sedghi LM (2021). Front Cell Infect Microbiol..

[2] Hu M (2025). Front Oral Health..

[3] Huoshen W (2025). Int Dent J..

[4] Mira A (2017). J. Clin. Periodontol..

[5] Tribble GD, Lamont RJ (2010). Periodontol..

[6] Zhou W (2016). PLoS One..

[7] Thamaraiselvan M, Jayakumar ND. (2024). J Adv Periodontol Implant Dent..

[8] Nakajima M (2025). Biomolecules..

[9] Amato M (2023). Pharmaceutics..

[10] Panwar M, Gupta SH (2009). Med. J. Armed Forces India..

[11] Mani K (2023). Pharmacognosy Reviews..

[12] Das AK (2009). J Non-Timber Forest Products..

[13] Dubey P (2025). Sci Rep..

[14] Sun JS (2002). Biomaterials..

[15] Vijay S (2022). Univ J Dent Sciences..

[16] Zeng H (2020). Med sci monit..

[17] Anuja GI (2010). J Ethnopharmacol..

[18] Pawar V (2017). IOSR Journal of Dental and Medical Sciences..

[19] Marimuthu J (2022). Ferns..

[20] Deepa N (2025). Journal of Pharma Insights and Research..

[21] Tripathi KP (2025). Bioinformation..

[22] Behal R (2011). J Indian Soc Periodontol..

[23] Saini K (2021). World J Dent..

[24] Warad SB (2013). Anc Sci Life..

[25] Katariya C, Rajasekar A (2024). Cureus..

[26] Raghava KV (2019). J Contemp Dent Pract..

[27] Singh S (2021). J Pharm Bioallied Sci..

[28] Van Strydonck DAC (2012). J Clin Periodontol..

[29] James P (2017). Cochrane Database Syst Rev..

